# Prevalence of MDM2 amplification and coalterations in 523 advanced cancer patients in the MD Anderson phase 1 clinic

**DOI:** 10.18632/oncotarget.26075

**Published:** 2018-09-04

**Authors:** Vikas Dembla, Neeta Somaiah, Pedro Barata, Kenneth Hess, Siqing Fu, Filip Janku, Daniel D. Karp, Aung Naing, Sarina Anne Piha-Paul, Vivek Subbiah, Apostolia M. Tsimberidou, Kenna Shaw, Funda Meric-Bernstam, David S. Hong

**Affiliations:** ^1^ Department of Investigational Cancer Therapeutics (Phase 1 Program), The University of Texas MD Anderson Cancer Center, Houston, Texas, USA; ^2^ Department of Sarcoma Medical Oncology, The University of Texas MD Anderson Cancer Center, Houston, Texas, USA; ^3^ Department of Solid Tumors, Taussig Cancer Institute, Cleveland Clinic, Cleveland, Ohio, USA; ^4^ Department of Biostatistics, The University of Texas MD Anderson Cancer Center, Houston, Texas, USA; ^5^ Sheikh Khalifa Bin Zayed Al Nahyan Institute for Personalized Cancer Therapy, The University of Texas MD Anderson Cancer Center, Houston, Texas, USA

**Keywords:** MDM2 amplification, phase I trials, solid tumors, TP53 mutation, CDK4 amplification

## Abstract

**Background:**

*TP53* is the most commonly mutated gene in cancer and codes for the best studied tumor suppressor, p53. MDM2 is involved in the negative regulation of p53 and itself serves as an oncogene, reported to be overexpressed in several cancer tumor types. In this retrospective study, we assessed the occurrence of *MDM2* amplification among patients with various types of cancers and its association with clinical factors, other genetic aberrations, and response to targeted therapy in a phase I clinical trial setting.

**Methods:**

Samples from patients with advanced solid tumors who had been referred to the MD Anderson phase I clinical trials program between January 2011 and January 2016 were collected and analyzed for *MDM2* amplification using FoundationOne’s genomic profiling assay. Patients whose tumors expressed *MDM2* amplification were compared to those with tumors of the same histologic types without *MDM2* amplification.

**Results:**

We tested tumors from 523 patients, of which 23 (4.4%) had *MDM2* amplification. The highest prevalence of *MDM2* amplification was in sarcoma (57%), breast cancer (13%) and bladder cancer (9%). Six patients with liposarcoma were treated on phase I protocol with an MDM2 inhibitor. The most common molecular aberrations co-occurring with *MDM2* amplification was *CDK4* amplification (70%). *TP53* mutation was also detected in 7 patients (30%).

**Conclusion:**

*MDM2* amplification was most commonly associated with liposarcoma. Concomitant alterations in additional genes such as *CDK4* amplification and *TP53* mutations, along with variable responses to targeted therapies including MDM2 inhibitors, suggest that further combinational studies are needed to target this population.

## INTRODUCTION

The tumor protein p53 (hereafter, p53) is one of the most studied tumor suppressors, with *TP53* being the most frequently mutated gene in cancer [1]. The p53 pathway is responsible for sequence-specific transcriptional activation (both *in vivo* and *in vitro*)[2, 3] and perturbations to this pathway are present during the development of most cancers [4]. Since p53 currently cannot be directly targeted by drugs, the focus of therapeutic strategies instead is on negative regulators of the *TP53* gene.

The murine double minute 2 (*MDM2*) gene was originally described in a tumorigenic mouse cell line because of its amplification [5, 6]. The human homolog of *MDM2* is mapped to chromosome 12q13-15 region [7, 8]. MDM2’s interaction with p53 and its negative regulation of p53 function was found to be mediated through two different mechanisms: 1) the direct binding of MDM2 to the *N*-terminal of p53, which subsequently inhibits the transcriptional activation function of p53, and 2) its activity as an E3 ubiquitin ligase, which targets p53 and facilitates its degradation through the 26S proteasome [9–11].

As a key negative regulator of p53 expression, MDM2 is thought to function as a proto-oncogene in preventing the accumulation of activated p53 [12]. This was verified in early studies showing *MDM2* overexpression in soft tissue sarcomas which was mutually exclusive with the occurrence of p53 mutations [13]. *MDM2* overexpression has since been reported in a variety of human tumors, mediated by either gene amplification or other mechanisms that remain unknown [14]. *MDM2* is known to be amplified or overexpressed in 40% to 60% of human sarcomas as well as in several other solid and hematological malignancies [15, 16]. Some tumors that overexpress *MDM2* also express wild-type p53, which is inactivated upon its interaction with MDM2 [17]. *MDM2* overexpression may be related to an increased likelihood of distant metastasis, decreased response to treatment, and poor clinical prognosis [14].

For these reasons, MDM2 has posed as an attractive and relevant target for cancer therapy. One strategy to promote p53 expression is to inhibit MDM2, since blocking the specific interaction between MDM2 and p53 would generate high levels of wild-type p53 and trigger apoptosis [18]. Releasing p53 may then contribute to cellular growth arrest and apoptosis. With this rationale, different MDM2-inhibiting molecules have been developed in recent years, and clinical trials of these agents are ongoing [15, 19].

Despite extensive data on MDM2 dysregulation in cancer, little is known about the characteristics of patients in whom *MDM2* gene expression is altered or amplified. With the emergence of MDM2 inhibitors, an understanding of this particular patient population would provide a better rationale for the use of these inhibitors and help establish criteria for selection of patients most likely to benefit from them. We therefore embarked on this retrospective study to assess the relationship between *MDM2* amplification and the clinical, pathologic and genetic characteristics of this *MDM2*-amplified patient population as well as their response to an MDM2 inhibitor in phase I trials, and from these provide a basis for further studies of the clinical role of MDM2 inhibitors.

## RESULTS

We identified 523 patients who had undergone FoundationOne testing for *MDM2* amplification and whose results were available for analysis. Of these, 23 (4.4%) had *MDM2* amplification in their tumor. As a comparison dataset, we identified another 124 of the 523 patients whose tumors did not have *MDM2* amplification or *MDM2* alterations but were of the same histologic types as those of the 23 *MDM2*-amplified patients. Selected demographic, diagnostic, and genetic characteristics of these two patient groups are summarized in Table 1.

**Table 1:** Patient characteristics and RMH score

Variable	Level	MDM2 Amplified	MDM2 NOT Amplified	p-value
	All Patients	N = 23	N = 124	
Mean Age	56 years	58 years	55 years	0.084^a^
Sex, n (%)	Female	10 (43)	75 (60)	0.13
	Male	13 (57)	49 (40)	
Race, n (%)	White	20 (87)	96 (81)	0.71
	Black	2 (9)	12 (10)	
	Other	1 (4)	11 (9)	
	Missing	0	5	
Tumor Type, n (%)	Sarcoma	13 (57)	20 (16)	<0.0001
	Breast	3 (13)	53 (43)	0.0071
	Bladder	2 (9)	8 (6)	0.69
	Liver	1 (4)	23 (19)	0.13
	Lung	1 (4)	10 (8)	1
	Salivary	1 (<1)	3 (<1)	
	Kidney	1 (<1)	3 (<1)	
	CUP	1 (<1)	4 (<1)	
Aberrations, n (%)	CDK4	16 (70)	2 (2)	<0.0001
	CDKN2A/B	5 (22)	6 (5)	0.015^b^
	MYC	5 (22)	21 (17)	0.56
	PIK3CA	1 (4)	19 (15)	0.2
	PTEN	0 (0)	16 (13)	0.077
	CCND1	1 (4)	13 (10)	0.7
	TP53	7 (30)	58 (47)	0.15^c^
RMH Score	0	11	67	0.42
	1	8	41	
	2,3	4	13	

Abbreviations: RMH, Royal Marsden Hospital score; CUP, carcinoma of unknown primary. RMH score could not be calculated in 4 patients in MDM2 not amplified group.

^a^ calculated using Wilcoxon rank sum test; ^b^ calculated using Fisher exact test. ^c^ calculated using chi-squared test.

The highest prevalence of *MDM2* amplification by histologic type was found in sarcoma patients (13 of 33 = 39% vs 10 of 114 non-sarcoma patients = 9%; p < 0.0001); the second highest was found in patients with metastatic breast cancer (3 of 56 = 5.3% vs 20 of 91 = 22%; p = 0.0071). Of the 13 sarcoma cases with *MDM2* amplification, liposarcoma was the most common histologic subtype (9 patients). The histologic subtypes, number of metastasis sites, performance status, and prior lines of therapy are summarized in Table 2. DNA samples were obtained for Foundation Medicine testing from all 523 patients prior to starting therapy.

**Table 2:** Histologic characteristics and status of patients according to MDM2 amplification status

Variable	MDM2 amplified (n = 23)	MDM2 not amplified (n = 124)
No. of metastatic sites		
0	0	1
1	6	42
2	9	53
3	5	23
4	3	5
ECOG Status		
0	2	6
1	19	116
2	2	2
No. of prior lines of therapy		
0	2	NA
1	4	NA
2	7	NA
3	7	NA
4	1	NA
5	0	NA
6	2	NA
Histologic Subtypes		
SARCOMA		
Liposarcoma	9	1
Osteosarcoma	2	11
Ewing sarcoma	1	4
Rhabdomyosarcoma NOS	1	4
BREAST CANCER		
Invasive Ductal carcinoma	2	32
NOS	1	14
Metaplastic	0	6
Adenocarcinoma	0	1
BLADDER CANCER (Urothelial carcinoma)	2	8
KIDNEY CANCER (Urothelial carcinoma)	1	3
UNKNOWN PRIMARY (Squamous Cell carcinoma)	1	4
LUNG CANCER		
Adenocarcinoma	1	9
NOS	0	1
SALIVARY GLAND CANCER		
Adenocarcinoma NOS	1	2
Adenoid cystic carcinoma	0	1
LIVER CANCER		
Hepatocellular carcinoma	1	19
Fibrolamellar	0	4

Abbreviations: ECOG, Eastern Cooperative Oncology Group; NOS, not otherwise specified; NA, not applicable.

The median albumin level was 4.1 g/dL (normal, 3.5 to 4.7 g/dL); the median lactate dehydrogenase level was 497 U/L (normal, 313 to 618 U/L); and the median number of metastatic sites was 2. The most common site of metastasis were the lungs (n = 57), followed by the bones (n = 48) and liver (n = 41). We also determined the Royal Marsden Hospital (RMH) score for patients included in our analysis (Table 1), with the exception of four (4) patients in the non-*MDM2* amplified group. This score is used to predict patient survival in phase I clinical trials; a lower score is generally associated with longer survival [20]. Among the 23 patients with *MDM2* amplification, 11 had an RMH score of 0 with a median overall survival (OS) was 24 months, which was significantly longer than the median OS of 6 months among the 12 patients who had an RMH score > 0 (hazard ratio [HR], 3.6; confidence interval [CI], 1.1, 11.5; p = 0.031). Among the non-*MDM2* amplified patients, 67 had an RMH score of 0 while the remaining patients had an RMH score > 0 ([HR] for MDM2 amplification adjusted for RMH = 0.6; [CI], 0.4, 1.1; p=0.13).

Sixteen of the 23 patients with *MDM2* amplification were noted to have co-occurrence of *CDK4* amplification, five had *CDKN2A/B* loss, and five had *MYC* amplification. Other aberrations that co-occurred with *MDM2* amplification in this analysis include mutations in *TP53* (7 patients), *PIK3CA* mutation (1), and *CCND1* amplification (1). Among the aberrations co-observed, *CDK4* amplification was most commonly noted in patients with soft tissue liposarcoma (9 of 16 patients), and *CDKN2A/B* loss was seen in one patient each with breast cancer not otherwise specified (NOS), adenocarcinoma of the lung, soft tissue liposarcoma, squamous cell carcinoma of unknown primary, and bladder urothelial carcinoma. *MYC* amplification was observed in patients with invasive breast carcinoma (2), breast carcinoma NOS (1), Ewing sarcoma (1), and bladder urothelial carcinoma (1). *TP53* mutation was seen in one patient each with kidney urothelial carcinoma, Ewing sarcoma, soft tissue liposarcoma, invasive ductal carcinoma of the breast, breast carcinoma NOS, hepatocellular carcinoma, and salivary adenocarcinoma NOS. No *TP53* mutation was noted in two patients with *MDM2* amplified bladder cancer.

For the 23 patients with *MDM2* amplification, we determined the copy numbers (CNs), as determined via next-generation sequencing by FoundationOne: total, 6-150 CNs; mean, 30.39; and median, 16. The highest CNs were in patients with soft tissue liposarcoma (29-150 CNs). The lowest were in patients with liver cancer (6 CNs), unknown primary cancer (7 CNs), breast cancer (7 CNs), and lung cancer (9 CNs).

Of the three breast cancer cases, two were estrogen receptor (ER)-positive, but were progesterone receptor-negative. One of the two ER-positive tumors also had *Her2* amplification (by fluorescence *in situ* hybridization). Two of the three tumors had *TP53* mutation, and all three had *MYC* amplification.

**Figure 1:**
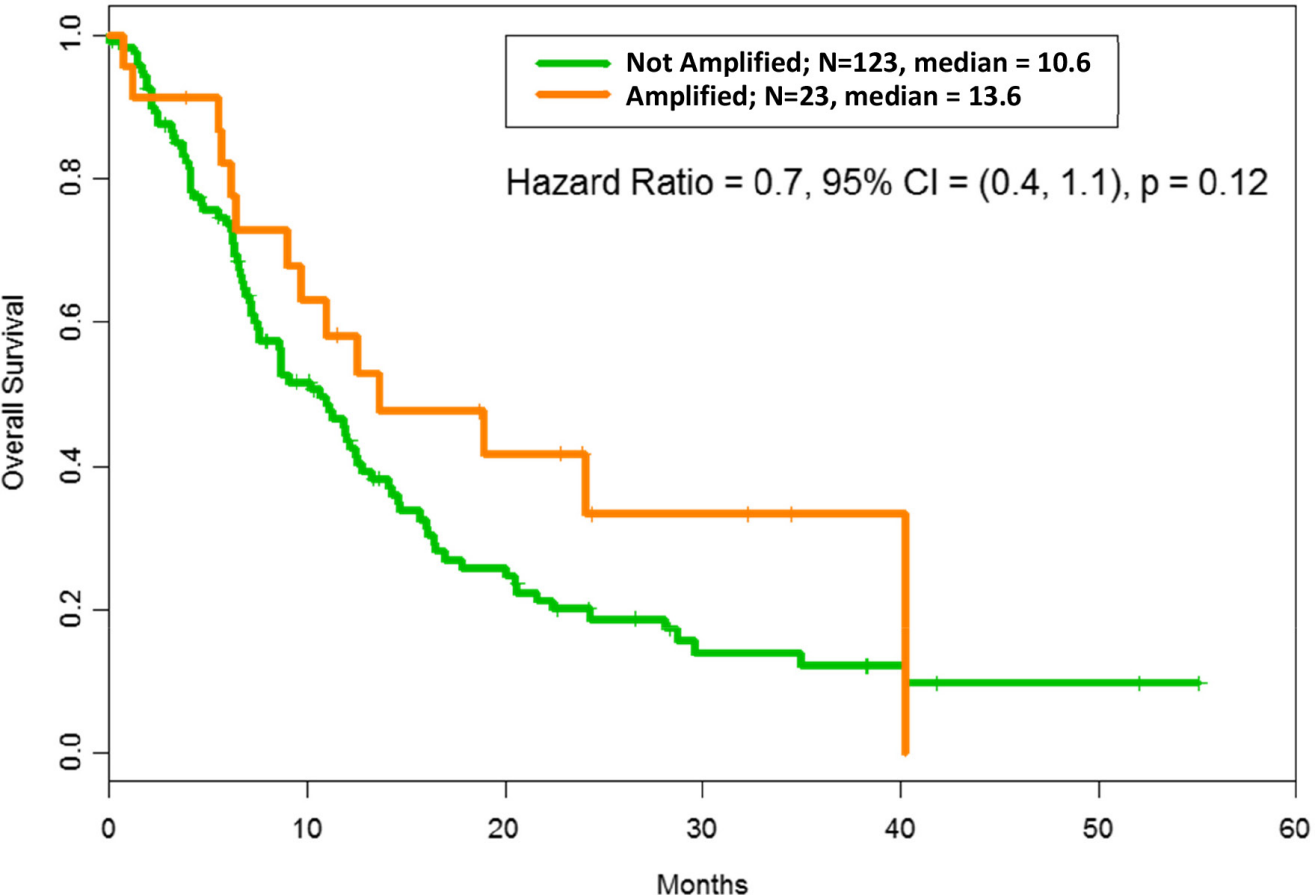
Overall survival according to MDM2 amplification status: After adjusting for RMH score, OS with MDM2 amplification = 13.6 months vs OS without MDM2 amplification= 10.6 months, hazard ratio = 0.6, confidence interval (CI) = (0.4, 1.1); p = 0.12. The median refers to months of survival

As shown in Figure 1, the median OS in patients with *MDM2* amplification was 13.6 months versus 10.6 months in patients without amplification (p = 0.12). Survival duration could not be accurately determined for one patient without *MDM2* amplification; hence, the median OS is reported for 123 patients. The median overall age was 56 years (range = 15-81 years); the median age of patients with *MDM2* amplification was 58 years (range = 23-77 years); and the median age of patients without *MDM2* amplification was 55 years (range = 15-81 years). In a Cox proportional hazard model with age > 60 years, the adjusted hazard ratio for *MDM2* amplification was 0.59 (range = 0.33-1.04; p = 0.068), and the adjusted hazard ratio for age > 60 years was 1.50 (range = 1.01-2.24; p = 0.045). Of the 124 patients without MDM2 amplification, 53 (43%) patients had breast cancer while of the 23 patients with MDM2 amplification, 3 (13%) had breast cancer; the Fisher exact test p-value for breast cancer was 0.0090. The p-values for the co-amplifications were also Fisher exact test p-values and therefore valid for small numbers. The median follow-up period in all 147 patients was 28.3 months, with 104 deaths observed.

Six of the 23 patients with *MDM2* amplification were enrolled in a trial involving an MDM2 inhibitor. All six patients had liposarcoma. There was a partial response in three patients and stable disease in two patients (15.7 months and 4.7 months). The sixth patient was still on trial at the time of this analysis. *TP53* mutation was noted in one of these six patients (this patient had enrolled prior to the availability of FoundationOne results). None of the 23 patients with *MDM2* amplification was treated with prior immunotherapy.

## DISCUSSION

To our knowledge, this study is the largest analysis of *MDM2* amplification in solid tumors reported in the current literature. We found a low incidence (4.4%) of *MDM2* amplification among 523 patients whose solid tumors were sent for next-generation sequencing testing with Foundation One. *MDM2* amplification was most frequent in patients with liposarcoma, followed by patients with metastatic breast cancer. Other aberrations that most frequently co-occurred with *MDM2* amplification were *CDK4* amplification, *CDKN2A/B* loss, and *MYC* amplification. Concomitant alterations of *MDM2* and *CDK4* are known and have been described in liposarcoma [8, 21, 22]. Previous studies have suggested that the presence of neochromosomes in cancer and sarcoma in particular, could explain the mechanism of *MDM2* amplification in these tumors. Neochromosomes, which are extra chromosomal structures that harbor oncogenes at high copy numbers, could incorporate Chr12q fragments including *MDM2,* and drive tumorigenesis [23]. Interestingly, we also found that *TP53* mutations and *MDM2* amplification were not mutually exclusive in our analysis.

Given the prevalence of co-occurrence of *MDM2/CDK4* amplification, targeting tumors that harbor these aberrations, combination therapy incorporating inhibitors of these two amplifications is an attractive proposition. Among the six patients with liposarcoma, histology was noted to be atypical lipomatous tumor in three patients and dedifferentiated liposarcoma in the remaining three patients. Currently, there are only two clinical trials evaluating the combination of an MDM2 inhibitor and a CDK4 inhibitor (ClinicalTrials.gov identifiers NCT02343172 and NCT01692496). Both of these trials are in liposarcoma patients, not breast cancer patients. Grünewald et al [24] reported co-expression of CDK4 amplification with *MDM2* amplification in one patient with salivary duct carcinoma. In another analysis, Roshida et al [25] reported an overall co-occurrence rate of 6.5% in 107 osteosarcoma patients whose tumors were tested for *MDM2/CDK4* amplification. This co-occurrence was associated with a low grade of osteosarcoma.

The significance of our study lies in the larger number of patients tested for *MDM2* amplification in our analysis compared to what has been previously reported. Previous studies report an incidence of *MDM2* amplification from 0% to 6.3%[26]. In our study, we report 4.4%, which is consistent with earlier reported studies. Interestingly, all six patients who were enrolled in an MDM2 inhibitor trial in our study had liposarcoma. The best response was a partial response in three patients and stable disease in two patients; the sixth patient was still undergoing therapy on a trial at the time of the analysis. It is difficult to make comparisons given our small numbers of liposarcoma patients but these results compare favorably to prior data from Livingston et al [27].

While the number of patients with *MDM2* amplification was too small to be meaningful in our analysis, there was a trend toward longer OS in the 23 patients whose tumors had *MDM2* amplification.

Three of the 23 (13%) patients with *MDM2* amplification had a diagnosis of metastatic breast cancer. Despite the high percentage in this very limited series, two of these three patients harbored mutant *TP53* and therefore were not eligible for enrollment in trials involving an MDM2 inhibitor. Most MDM2 inhibitors under development target the MDM2-p53 complex, including Nutlin-3 [18], RITA [28], MI-219 [29], AMG232 [30], and SAR405838 [31]. These inhibitors have little or no effect on cancers with mutant *TP53*. Selective inhibitors of ubiquitin-specific protease-7 (USP7) such as GNE-6640 and GNE-6776 are also being studied as potential stabilizers of p53 via MDM2 [32]. USP7 regulates stability of p53 tumor suppressor and other proteins necessary for tumor cell survival. GNE-6640 and GNE-6776-like molecules induce tumor cell death and enhance cytotoxicity with chemotherapeutic agents and targeted compounds. This strategy may be broadened for developing other deubiquitinase inhibitors to inhibit proteins that require ubiquitin binding for full functional activity. Chrisanthar et al [33] reported that patients with stage III breast cancer with the MDM2 SNP309 genotype did not experience a response to either epirubicin or paclitaxel. The frequency of mutant *TP53* in breast cancer has been reported to be 30% to 73%, but it fluctuates widely between subclasses of breast cancer [34, 35]. As a result, MDM2 inhibitors have no significant anticancer activity in such tumors. Therefore, new strategies to target MDM2 are needed. Indeed, new studies are underway to develop a new class of MDM2 inhibitors that exhibit anticancer activity, regardless of the tumor’s p53 status [36].

One very interesting finding in our analysis was the presence of mutant *TP53* in seven patients. This was not significantly different from the rate in patients without *MDM2* amplification (30% vs 47%, p=0.15). Nonetheless, the co-existence of mutant *TP53* with MDM2 amplification is surprising, as they have been thought to be alternative mechanisms for inactivating the suppressing cell growth pathway and thus mutually exclusive [37]. A recent study by Sanchez-Vega et al [38] reports the mutual exclusivity of *TP53* mutations and *MDM2* amplification in tumors profiled by The Cancer Genome Atlas (TCGA). The discrepancy between our results and this recent report may be largely due to the nature of TCGA curated samples which are largely from primary tumors, whereas our study includes metastatic tumor samples from heavily pretreated patients.

Tumors with mutant *TP53* are canonically thought to not respond to MDM2 inhibition. Our analysis clearly shows that this is not a mutually exclusive phenomenon and that subsets of patients with MDM2 amplification also have a co-existing mutant *TP53*. This conflicting observation is uncommon but definitely has been described in the literature [37, 39–42]. Florenes et al [39] related *MDM2* amplification to *TP53* status. *MDM2* amplification was noted in ten tumors (10.3%), while *TP53* mutation was noted in 12. However, only one case had both *TP53* mutation and high levels of *MDM2* mRNA. In a study by Cordon-Cardo et al [40], 211 adult soft tissue sarcomas were studied to detect molecular abnormalities of *TP53* and *MDM2* expression. Monoclonal antibodies directed against MDM2 and p53 proteins were used to measure their overexpression. Strikingly, 22 cases had high levels of both p53 and MDM2 proteins on the basis of immuno-reactivity; this finding was statistically significant (p < 0.05) and associated with poor survival. However, overexpression of MDM2 and p53 proteins in the nuclei of tumor cells is not correlated with *MDM2* gene amplification or *TP53* mutation. Co-occurrence of *MDM2* amplification and *TP53* mutation was also noted in two patients by Grünewald et al [24]as well. Drummond et al [42] reported that *TP53* mutant, *MDM2* amplified cell lines that were resistant to MDM2 inhibitors retain sensitivity to ionizing radiation and suggested that such patients may have alternative treatment options like radiation therapy. Saiki et al [43] demonstrated that *MDM2* amplification and *TP53 mutation* are not mutually exclusive in tumor cell lines, possibly because of a misidentified *TP53* mutation or heterozygous *TP53* mutation or because cell lines harbor viral gene sequences known to inactivate p53. It is also possible that MDM2 affects p53 in a dose-dependent manner, and only tumors that carry high copy numbers of the *MDM2* gene express enough protein to inhibit p53 [41]. Co-existence of both these aberrations should also be expected if both events are independent of each other. On the basis of this observation and our results which also showed that *MDM2* amplification and *TP53* mutations are not mutually exclusive in advanced cancers, alteration in one gene should not preclude testing for the other. It is important to check for the *TP53* mutation status once *MDM2* amplification has been reported in a next-generation sequencing test.

The reported incidence of *MDM2* amplification in various series conducted in single tumor types (0% to 6.3%) is consistent with our findings [24, 26, 44, 45]. Grünewald et al [24], in a series of 51 patients with salivary duct carcinomas, reported *MDM2* amplification in three patients (5.8%), as well as synchronous *CDK4* and *MDM2* amplification in one patient with a co-expression rate of 33.3%. We observed a higher co-expression rate in our study (16 of 23 [(70%]). In addition, our analysis included a much larger patient cohort. Michalk et al [26] reported a 6.3% incidence of *MDM2* amplification in esophageal carcinomas (adenocarcinoma and squamous cell carcinoma), and Schoolmeester et al [44] found an incidence of 5% (2 of 43 cases) in endometrial stromal tumors. In contrast, Lyle et al [45] found no *MDM2* amplification in their series of ten tests among 38 patients with malignant phyllodes tumor of the breast. Zhu et al [46] reported the first documented cases of *MDM2* amplification in laryngeal and hypopharyngeal liposarcoma. In a separate study by Kato et al [47], *MDM2* has been recognized as a marker of pseudo-progression in patients treated with single-agent checkpoint (PD-1/PDL-1) inhibitors. None of the 23 patients with *MDM2* amplification in our analysis was treated with prior immunotherapy as of the cut-off date. Hence, there is no realistic way to define this correlation in our analysis.

Our analysis constitutes, to our knowledge, one of the largest series of solid tumors tested for *MDM2* amplification at a single center. We do recognize that it is not without its limitations. For instance, our study was a retrospective analysis and is hence prone to selection bias. We were also restricted to disclose the identity of the MDM2 inhibitors used in our analysis. We could not validate positive *MDM2* amplification results using fluorescence *in situ* hybridization or immunohistochemical analysis. Because *MDM2* amplification was analyzed in multiple tumor types, the number of cases with *MDM2* amplification per tumor type is small for any meaningful analysis of correlations. Some gene alterations that co-occurred with MDM2 amplification in our analysis did not meet statistical significance and the probability that these represent chance events cannot be excluded. We refrained from making comparisons between our analysis and the TCGA dataset since it would not have been accurate—as mentioned earlier, the TCGA dataset is mainly comprised of samples from patients in the earlier stages of cancer, whereas our patient population in consisted mainly of metastatic, recurrent and pre-treated patients. Regardless of these limitations, we found that *MDM2* amplification in solid tumors was associated with liposarcoma, metastatic breast cancer, *CDK4* amplification, *TP53* mutation, *CDKN2A/B* loss, and *MYC* amplification.

Our finding that *MDM2* and *CDK4* amplification were often co-expressed suggests that therapeutic strategies combining MDM2 and CDK4 inhibitors might have promise in patients whose tumors express both of these aberrations. Further studies are necessary to better identify and treat these patients. Moreover, our results also emphasize the importance of assessing the *TP53* mutation status in all patients found to have *MDM2* amplification.

## MATERIALS AND METHODS

### Patients

We retrospectively reviewed the electronic medical records of patients with solid tumors who were referred to the Department of Investigational Cancer Therapeutics at The University of Texas MD Anderson Cancer Center, Houston, Texas from January 2011 through January 2016 and for whom results of a clinical next-generation sequencing-based assay (Foundation One) were available. Patients were eligible for inclusion if their malignancy had been histologically confirmed by the Department of Pathology at MD Anderson. Data were collected from transcribed notes and radiology reports in the electronic medical record and other sources of documentation. All patients had been registered in the institutional patient database, and all clinical, pathologic, and laboratory assessments had been performed at the institution. The study and all treatments were conducted according to the guidelines and with the approval of the Institutional Review Board.

### Tissue samples and molecular analysis

We analyzed 523 clinical cases with various disease origins using comprehensive genomic profiling in a Clinical Laboratory Improvement Amendments-certified, College of American Pathologists-accredited laboratory (Foundation Medicine, Cambridge, MA, USA). The pathologic diagnosis of each case was confirmed by routine hematoxylin and eosin staining, and all samples forwarded for DNA or RNA extraction contained a minimum of 20% tumor nuclei. Extensive technical descriptions and validation of the genomic profiling assays used to analyze these samples in the course of clinical care have been published previously [48, 49]. In summary, 50 nano grams of DNA was extracted from 40 microns (10 x 4 microns) thick cuts of tumor sample from formalin-fixed, paraffin-embedded tissue blocks or slides. Targeted next-generation sequencing was performed on hybridization-captured, adaptor ligation-based libraries for all coding exons of at least 236 cancer-related genes, including MDM2 gene, plus select introns from at least 19 genes that are frequently rearranged in cancer. For those samples for which RNA was available, targeted RNA sequencing was performed for enhanced rearrangement analysis in 265 genes [49]. Sequencing of captured libraries was performed using the Illumina HiSeq 2000 or HiSeq 2500 platforms to a mean exon coverage depth of 1164X, and the resultant sequences were analyzed for base substitutions, insertions, deletions, copy numbers (CNs) alterations (focal amplifications and homozygous deletions), and select gene fusions.

### Treatment and evaluation

Six patients in the study cohort were enrolled in a phase I clinical trial involving MDM2 inhibitor. This enrollment occurred at the discretion of treating physicians and per the availability of an appropriate clinical trial. Treatment was continued until the onset of disease progression or unacceptable toxicity, according to the specific treatment protocol for each trial. Clinical assessments were performed as specified in each protocol, typically before the initiation of therapy and then at the beginning of each new treatment cycle. Response was assessed from computed tomography scans at baseline before treatment initiation and then every 2 cycles during the first 6-8 cycles. All images were read in the Department of Radiology and were reviewed by the Department of Investigational Cancer Therapeutics tumor measurement clinic. Responses were categorized according to RECIST 1.1 (Response Evaluation Criteria in Solid Tumors) criteria on the basis of specific protocol requirements and were reported as the best response [50, 51]. We also calculated the Royal Marsden Hospital (RMH) score, which is a prognostic score based on 3 survival-associated objective markers (serum albumin level, serum lactate dehydrogenase level, and number of metastasis sites). The RMH score was developed in 2009 to improve patient selection for phase I clinical trials [20].

### Statistical analysis

Cox proportional hazards regression analysis was used to estimate hazard ratios along with corresponding confidence intervals and p-values. Survival curves were estimated using the Kaplan–Meier method. Median survival time was estimated as the point at which the survival curve crossed 50%. The proportions between groups were compared by chi-squared tests or Fisher exact tests, as appropriate for the data.

Patient characteristics, including demographics, tumor type, tumor MDM2 amplification status, and associated genetic abnormalities, were summarized by descriptive statistics. All statistical analyses were carried out using S+ 8.2 for Windows (TIBCO Software, Inc., Palo Alto, CA, USA).

## Abbreviations

OS: Overall Survival
ECOG: Eastern Cooperative Oncology Group
NOS: not otherwise specified
NA: not applicable
RMH: Royal Marsden Hospital score
CUP: carcinoma of unknown primary
